# Species-Level Associations of Leaf Traits and Photosynthetic Capacity in Common Woody Plants of Subtropical Secondary Forest Vegetation

**DOI:** 10.3390/plants15213295

**Published:** 2026-10-25

**Authors:** Manyi Li, Lei Ma, Yunqi Wang, Lu Yao, Mingze Xu, Cheng Li

**Affiliations:** 1 Observation and Research Station of Ecological Restoration for Chongqing Typical Mining Areas, Ministry of Natural Resources ( Chongqing Institute of Geology and Mineral Resources), Chongqing 401120, China; limanyi@cqdky.com (M.L.); malei@cqdky.com (L.M.); zhaokun001223@bjfu.edu.cn (L.Y.); xumingze@cqdky.com (M.X.); 2 College of Resources and Safety Engineering, Chongqing University, Chongqing 400044, China; 3 Thre Gorges Reseroir Area (Chongging) Forest Ecosysten Research Station, School of Soil and Water Conservation, Beijing Forestry University, Beijing 100083, China; wangyunqi@bjfu.edu.cn

**Keywords:** leaf traits, photosynthetic capacity, species-level analysis, nitrogen content per unit leaf area, mathematical coupling, light-response curves

## Abstract

Leaf functional traits are widely used to describe interspecific variation in plant resource-use strategies and photosynthetic performance. We examined leaf traits and photosynthetic characteristics in 21 common woody species (11 tree and 10 shrub species; two individuals per species) sampled from subtropical secondary-forest vegetation in Chongqing, southwestern China. Maximum net photosynthetic rate (Pnmax), apparent quantum yield (AQY), light saturation point (LSP), and light compensation point (LCP) were derived from species-level fitted light-response curves. Among the 17 species with accepted fits, Pnmax ranged from 3.35 to 12.93 μmol m^−2^ s^−1^. Estimates for *Jacaranda mimosifolia* and *Vitex negundo* var. *cannabifolia* were extrapolated beyond the measured irradiance range (14.66 and 16.23 μmol m^−2^ s^−1^, respectively). No finite Pnmax was defined for *Osmanthus fragrans* or *Rhododendron simsii* because β was estimated at zero; their fitted asymptotic net photosynthetic rates were 3.33 and 4.29 μmol m^−2^ s^−1^, respectively. Nitrogen content per unit leaf area (Narea) ranged from 0.46 to 2.28 g m^−2^. Trait analyses used species means as independent observations. Spearman correlations were evaluated with Benjamini–Hochberg false discovery rate correction, and prespecified candidate regression models were compared using adjusted R^2^, AICc, and leave-one-species-out cross-validation. After FDR correction, Narea was positively associated with Pnmax (Spearman’s ρ = 0.673, q = 0.0137) and AQY (ρ = 0.666, q = 0.0137). No statistically significant differences in photosynthetic parameters remained between trees and shrubs after correction for multiple testing. Among the prespecified candidate models, the Narea-only model had the lowest AICc for Pnmax (adjusted R^2^ = 0.440). For AQY, the Narea-only and Narea + LA models had similar support (ΔAICc = 0.41), and the data did not clearly distinguish between them. Adding LA did not significantly improve either model in the incremental F-tests (*p* = 0.486 for Pnmax and *p* = 0.135 for AQY). The positive Pnmax–Narea association remained after controlling for LMA using partial Spearman correlation (partial ρ = 0.762, *p* < 0.001), indicating that shared LMA normalization alone did not explain the association in this dataset. These species-level associations are observational and do not establish causation.

## 1. Introduction

Amid intensifying global climate change and ongoing biodiversity loss, studying plant leaf functional traits and their environmental adaptability has become increasingly important [1,2]. Functional plant traits are widely utilized to evaluate biodiversity conservation efforts and ecosystem management effectiveness [3]. Accurately identifying and quantifying the relationships among these traits is crucial for understanding their roles in plant physiological and ecological processes. As the primary site of photosynthesis, leaf traits are closely correlated with various physiological functions in plants [4] and exhibit high plasticity [5,6]. Leaf functional traits and ecological stoichiometric characteristics provide insights into plant adaptation strategies, self-regulation mechanisms, and resource allocation patterns [7]. Leaf functional traits, including photosynthetic parameters, SLA, LDMC, and Narea, have been widely used to characterize interspecific variation in plant resource-use strategies and leaf economics spectra [8,9,10,11]. Leaf traits and photosynthetic performance can vary with plant water status and leaf thermal conditions [12,13,14]. Analyzing the relationships among leaf traits allows for the inference of difficult-to-measure traits from those that are easier to quantify [15]. However, whether a universal model can quantitatively predict these relationships remains uncertain. Therefore, further research on the quantitative relationship among leaf traits is essential for understanding trait variation, scaling laws, and constraints on plant-atmosphere carbon exchange [15].

The interdependence of leaf traits underscores the complexity of photosynthesis as a physiological process influenced by multiple factors [16], with its characteristic parameters and environmental factors jointly determining plant photosynthetic efficiency [17]. Light response curves, a crucial tool in physiological ecology research [18,19], illustrate the relationship between photosynthetic rate and light intensity, and they are widely utilized to evaluate plant adaptability to environmental changes. These parameters not only indicate plant resilience and stress responses but also play a pivotal role in determining plant growth and productivity [20], making them significant in photosynthetic trait studies [21].

Numerous leaf traits are strongly associated with photosynthesis, with variations in structural and biochemical characteristics reflecting the diversity of plant carbon assimilation capabilities. This provides a theoretical basis for predicting photosynthetic performance under varying environmental conditions. For example, some plants enhance photosynthetic rates by expanding leaf area to optimize light capture efficiency [22], while chlorophyll content also rises with increasing leaf area. Conversely, reducing leaf area or increasing LDMC can mitigate water loss due to transpiration [23,24]. Leaf nitrogen is distributed among Rubisco, other Calvin-cycle enzymes, thylakoid proteins, and non-photosynthetic compounds. The photosynthetic apparatus collectively represents a substantial fraction of leaf nitrogen, although the exact allocation varies among species and environmental conditions [25,26]. Trait values can differ between controlled and field measurements, underscoring the need for cautious interpretation of trait-based associations [27]. Morphological traits, including leaf area (LA) and LDMC, provide insights into plant resource use efficiency and photosynthetic potential [28], with LA variations closely correlated with light capture and carbon assimilation capacity [6]. SLA, defined as the ratio of LA to leaf dry mass, directly reflects plant light capture efficiency and carbon assimilation strategies [29,30]. Studies show that changes in LA and LDMC are strongly associated with plant growth forms [31], while large-scale research highlights plant life forms and functional types as primary factors influencing leaf trait variation [32]. However, current international research on leaf photosynthesis predominantly focuses on light reactions and carbon metabolism, with limited quantitative studies on the influence of structural factors. Most studies examine correlations between environmental conditions and leaf traits [33] or focus on individual species or traits [34,35]. Despite their crucial role in plant adaptation to environmental changes, photosynthetic characteristics remain understudied [36,37]. Plants of different growth types adapt to their environments through distinct physiological and ecological traits, thereby enhancing resource use efficiency [38,39]. A detailed examination of leaf morphological, chemical, and physiological traits provides valuable insights into the mechanisms underlying plant adaptation to environmental changes [30]. Photosynthetic capacity may be associated with both leaf structural traits and nutrient investment. However, some area-based leaf traits are mathematically related. In particular, nitrogen content per unit leaf area (Narea) is calculated as mass-based nitrogen content (Nmass) multiplied by leaf mass per area (LMA), whereas LMA is the inverse of specific leaf area (SLA). Thus, Narea can also be expressed as Nmass/SLA. Associations involving Narea and SLA should therefore be interpreted cautiously and should not be regarded as evidence of independent or synergistic physiological mechanisms without additional independent measurements. This issue has been widely discussed in relation to mass-based and area-based expressions of leaf traits and the potential for normalization-induced correlations [40,41,42].

This study examined species-level associations between leaf traits and photosynthetic parameters in 21 common woody species sampled from subtropical secondary-forest vegetation in Chongqing, southwestern China. We addressed the following questions: (1) How do photosynthetic parameters and leaf traits vary among the sampled woody species? (2) Are growth form, leaf habit, or their interaction associated with variation in these traits? (3) Which leaf traits are associated with variation in photosynthetic parameters at the species level? We expected substantial interspecific variation in leaf traits and photosynthetic parameters, whereas overall differences between tree and shrub growth forms would be limited after accounting for leaf habit. Because Narea is mathematically related to LMA and SLA, this potential coupling was recognized during reanalysis as a design constraint rather than treated as evidence of an independent biological mechanism.

## 2. Results

### 2.1. Analysis of Light Response Curve Parameters

**Figure 1:**
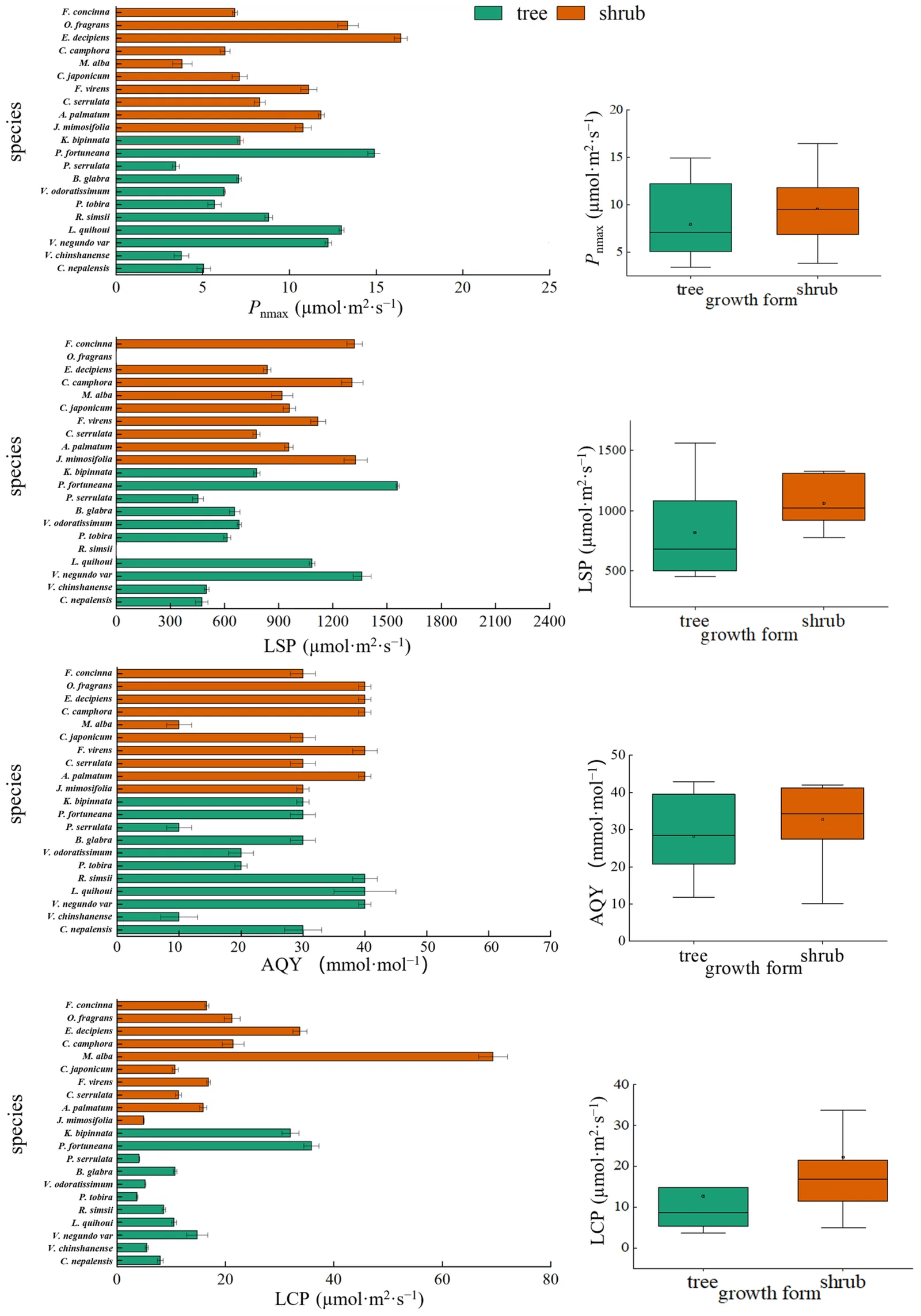
Interspecific variation and growth-form comparisons of photosynthetic light-response parameters in the sampled woody species. Pnmax, maximum net photosynthetic rate; AQY, apparent quantum yield; LSP, light saturation point; LCP, light compensation point. Bars show species means, and error bars indicate the standard deviation (SD) of repeated leaf-level measurements within each species; they are shown descriptively and were not treated as independent observations in inferential analyses. The figure is descriptive; results of the species-level tests for growth form, leaf habit, and their interaction are reported in the Section 2 and Table S4. For LSP, the boxplots summarize the 19 species with finite estimates (trees, *n* = 10; shrubs, *n* = 9), including two extrapolated estimates. No LSP value is shown for *Osmanthus fragrans* or *Rhododendron simsii* because β = 0 and their LSP was undefined.

For each species, repeated leaf-level Pn–I observations were averaged at each irradiance level before fitting one species-level modified rectangular hyperbola curve (Table 1). The light-response parameters reported below were derived from these species-level curves. Among the 17 species with accepted fits, Pnmax ranged from 3.35 to 12.93 μmol m^−2^ s^−1^. Estimates for *Jacaranda mimosifolia* and *Vitex negundo* var. *cannabifolia* were extrapolated beyond the measured irradiance range (14.66 and 16.23 μmol m^−2^ s^−1^, respectively). For *Osmanthus fragrans* and *Rhododendron simsii*, β was estimated at zero; therefore, LSP and a finite Pnmax were undefined, and the fitted curves approached asymptotic net photosynthetic rates of 3.33 and 4.29 μmol m^−2^ s^−1^, respectively. Empirical AQY, estimated as the slope of the linear regression using measured observations at nominal irradiances of 0, 30, 70, and 100 μmol m^−2^ s^−1^, ranged from 17.10 to 52.17 mmol mol^−1^. LCP ranged from 3.55 to 74.51 μmol m^−2^ s^−1^. Seventeen species had finite LSP estimates within the measured irradiance range (435.11–1314.90 μmol m^−2^ s^−1^); LSP estimates for *Jacaranda mimosifolia* and *Vitex negundo* var. *cannabifolia* were extrapolated. In the sensitivity analysis restricted to the 17 accepted LSP estimates, no trait–LSP correlation remained significant after FDR correction (all q ≥ 0.163). Fitted α values ranged from 0.0197 to 0.1099 mol mol^−1^ and were consistently larger than the empirical low-light AQY estimates; therefore, α was treated as an empirical curve-shape parameter, not as an independent biological estimate of AQY. After correction for multiple testing, no statistically significant differences between trees and shrubs remained for Pnmax, AQY, LSP, or LCP. Across species means, coefficients of variation (CVs) were 36.6% for Pnmax among the 17 accepted fits, 22.2% for AQY (*n* = 21), 31.5% for LSP among the 17 accepted estimates, 96.2% for LCP (n = 21), and 26.8% for fitted α (n = 21). Interspecific variation in these parameters is shown in Figure 1.

### 2.2. Analysis of Plant Leaf Functional Traits

**Figure 2:**
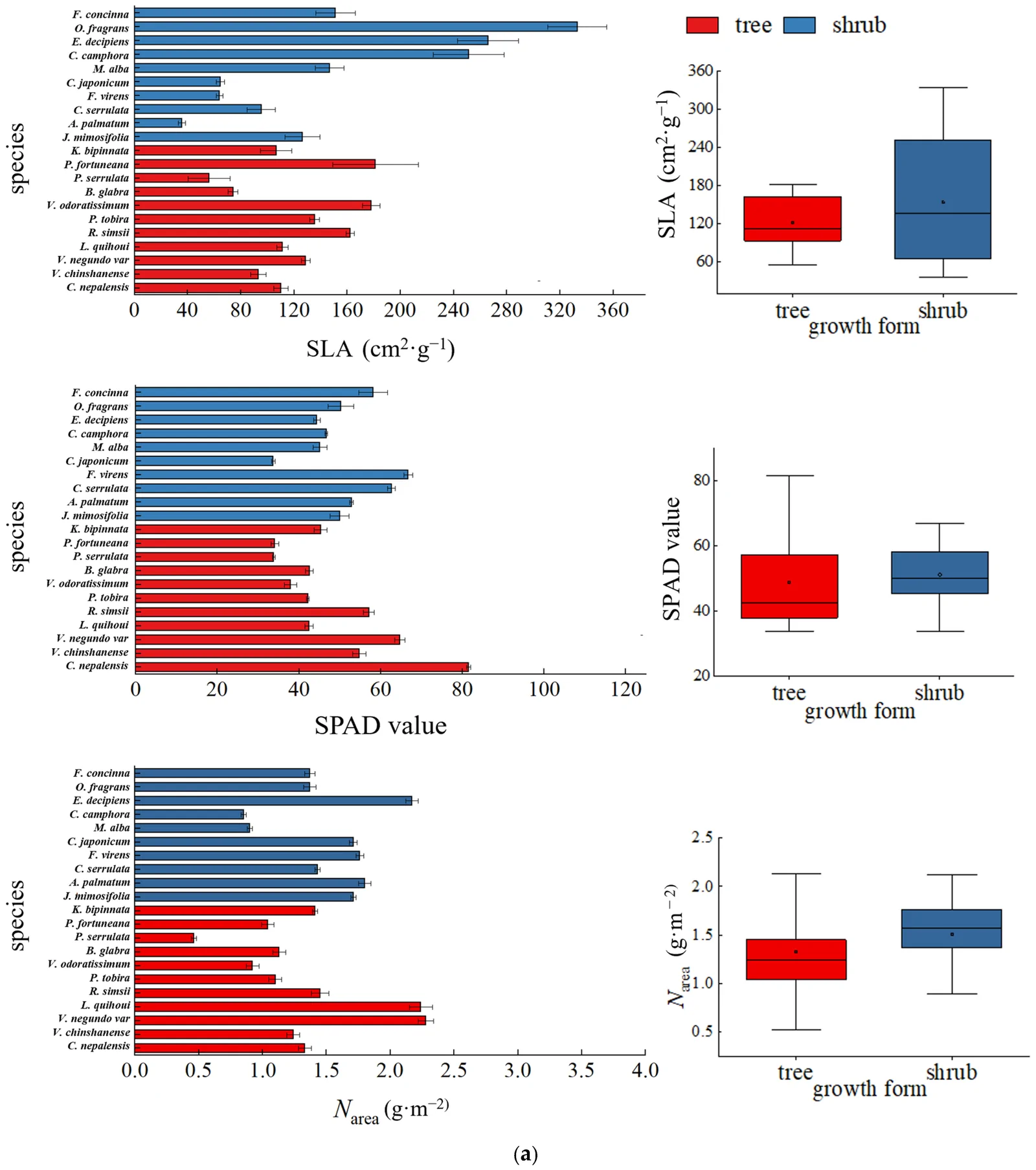

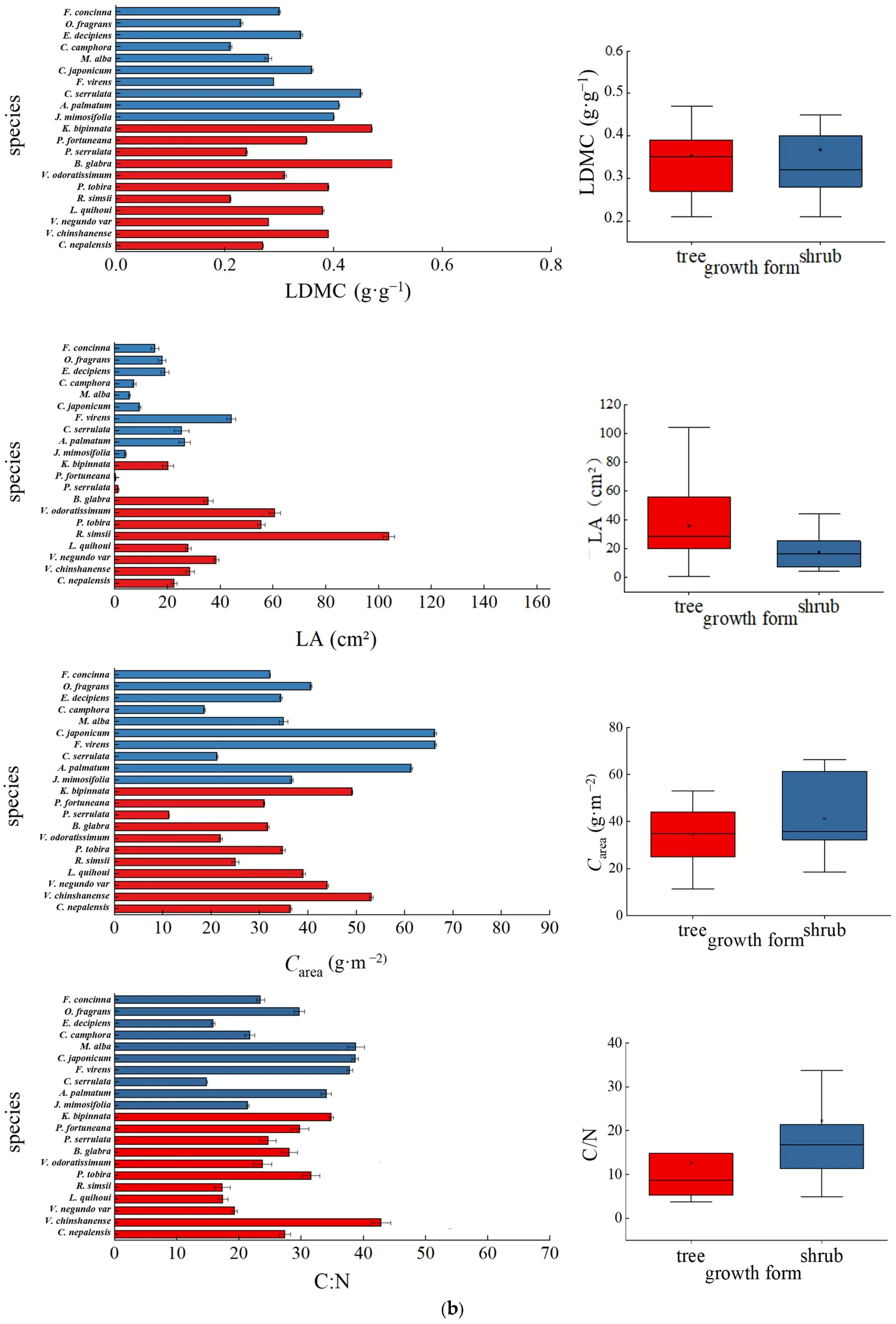
(**a**) Interspecific variation and growth-form comparisons of specific leaf area (SLA), SPAD value, and nitrogen content per unit leaf area (Narea) in the sampled woody species. Bars show species means, and error bars indicate the standard deviation (SD) of repeated leaf-level measurements within each species. Boxplots summarize the distribution of species means by growth form. The figure is descriptive; results of the species-level tests for growth form, leaf habit, and their interaction are reported in the Section 2 and Table S4. (**b**) Interspecific variation and growth-form comparisons of leaf dry matter content (LDMC), leaf area (LA), carbon content per unit leaf area (Carea), and carbon-to-nitrogen ratio (C:N) in the sampled woody species. Bars show species means, and error bars indicate the standard deviation (SD) of repeated leaf-level measurements within each species; they are shown descriptively and were not treated as independent observations in inferential analyses. The figure is descriptive; results of the species-level tests for growth form, leaf habit, and their interaction are reported in the Section 2 and Table S4.

Figure 2 illustrates variation in leaf functional traits among the 21 sampled woody species. Interspecific variation was observed in structural traits, including LA, LDMC, and SLA, as well as in biochemical and stoichiometric traits, including SPAD value, Carea, Narea, and C/N.

Overall, LA ranged from 0.37 to 103.87 cm^2^, LDMC ranged from 0.21 to 0.59 g g^−1^, and SLA ranged from 35.62 to 333.33 cm2 g^−1^. SPAD value ranged from approximately 33 to 81.50. Carea and Narea ranged from 11.22 to 66.33 g m^−2^ and from 0.46 to 2.28 g m^−2^, respectively, whereas C/N ranged from 14.80 to 42.81. Across species means (*n* = 21), CVs were 89.5% for LA, 27.7% for LDMC, 54.3% for SLA, 24.5% for SPAD value, 39.9% for Carea, 33.8% for Narea, and 30.6% for C:N.

Growth form, leaf habit, and their interaction were evaluated using species-level linear models. The three term-specific *p* values for each of the 11 response variables were adjusted together using the Benjamini–Hochberg false discovery rate procedure (33 tests). No main effect of growth form remained significant after FDR correction. For SLA, both the main effect of leaf habit (F1,17 = 12.729, raw *p* = 0.0024, q = 0.0391) and the growth form × leaf habit interaction (F1,17 = 14.134, raw *p* = 0.0016, q = 0.0391) were significant under the primary leaf-habit classification. Because the smallest growth form × leaf habit group contained only four species, the SLA interaction effect is interpreted as exploratory. No other growth-form, leaf-habit, or interaction effects remained significant after FDR correction. Complete results for the growth form, leaf habit, and interaction terms, including raw *p* values and FDR-adjusted q values, are provided in Table S4.

### 2.3. Correlation Analysis Between Light Response Curve Characteristic Parameters and Leaf Traits

**Figure 3:**
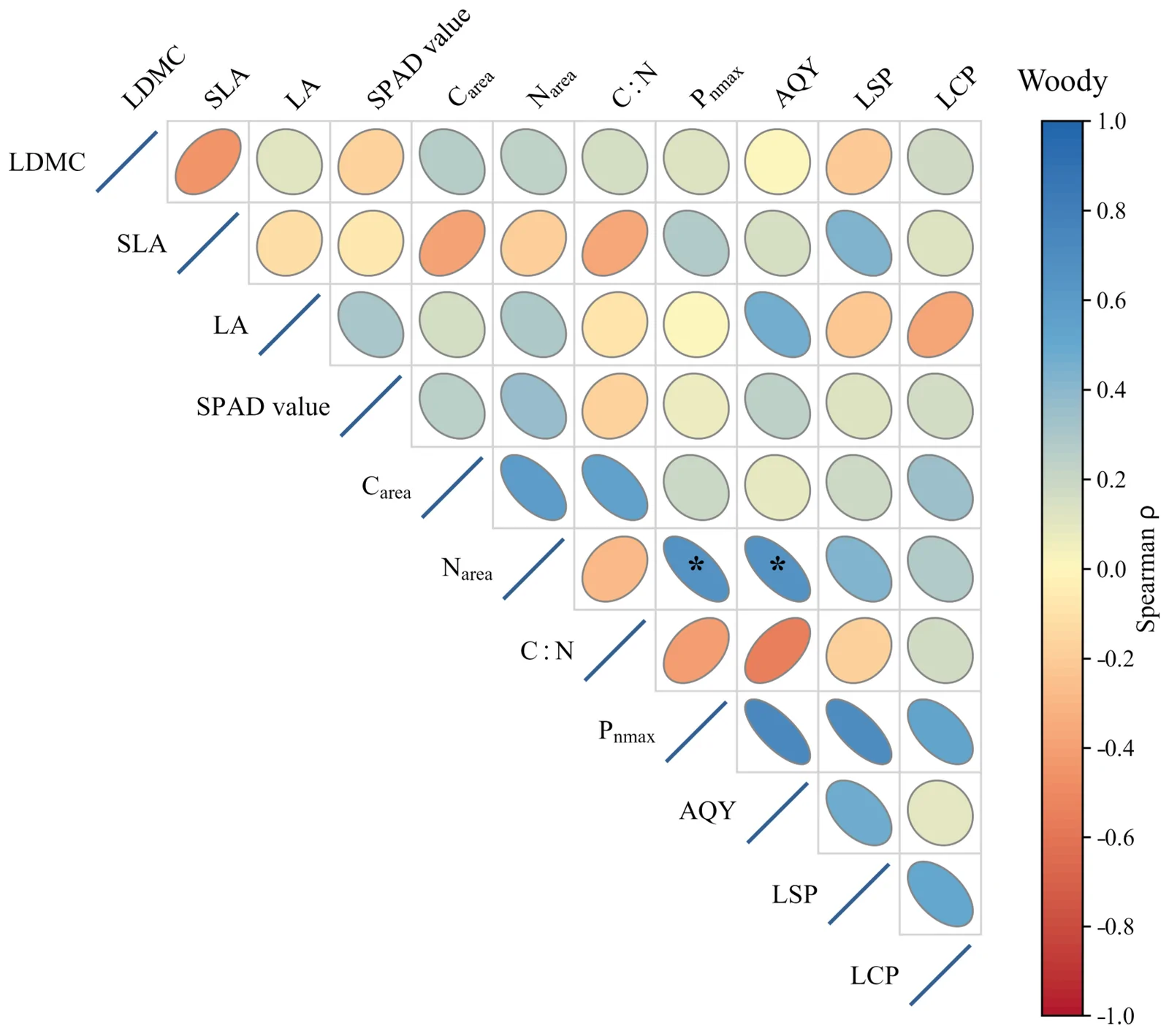
Spearman correlation matrix between leaf traits and photosynthetic parameters across the 21 sampled woody species, based on species means. Ellipses represent Spearman’s rho: blue and red indicate positive and negative correlations, respectively, and narrower ellipses indicate stronger correlations. Asterisks mark correlations that remained significant after Benjamini–Hochberg false discovery rate (FDR) correction across the 28 prespecified leaf-trait–photosynthetic-parameter correlations: Narea–Pnmax (rho = 0.673, q = 0.0137) and Narea–AQY (rho = 0.666, q = 0.0137). Numerical correlation coefficients are provided in Table S3. For correlations involving LSP, *n* = 19 because two species had no finite LSP estimate. Abbreviations: LDMC, leaf dry matter content; SLA, specific leaf area; LA, leaf area; SPAD value, SPAD meter reading; Carea, carbon content per unit leaf area; Narea, nitrogen content per unit leaf area; C:N, carbon-to-nitrogen ratio; Pnmax, maximum net photosynthetic rate; AQY, apparent quantum yield; LSP, light saturation point; LCP, light compensation point.

Spearman correlation analyses were conducted using species means for all 21 woody species. The 28 pre-specified trait–photosynthetic parameter correlations were treated as one family of tests, and *p* values were adjusted using the Benjamini–Hochberg false discovery rate procedure. After FDR correction, Narea was positively associated with both Pnmax (Spearman’s rho = 0.673, q = 0.0137) and AQY (Spearman’s rho = 0.666, q = 0.0137). No other correlations between the measured leaf traits and photosynthetic parameters remained significant after FDR correction. For correlations involving LSP, a sensitivity analysis restricted to the 17 accepted finite LSP estimates did not identify any significant trait–LSP association after FDR correction (all q ≥ 0.163). To assess the potential influence of LMA normalization, we examined both mass- and area-based relationships. Pnmax expressed on a mass basis was positively correlated with Nmass (Spearman’s ρ = 0.921, *p* < 0.001; Table S1); however, because both variables share LMA as a denominator, this correlation may be amplified by their common divisor and is not interpreted as independent corroboration. LMA was not significantly correlated with Narea (ρ = 0.187, *p* = 0.417) or area-based Pnmax (ρ = −0.265, *p* = 0.246), and Narea was not significantly correlated with SLA (ρ = −0.186, *p* = 0.420). A partial Spearman analysis controlling for LMA showed that the positive association between area-based Pnmax and Narea remained (partial ρ = 0.762, t (18) = 5.00, *p* < 0.001; *n* = 21). Together with the nonsignificant correlations of LMA with Narea and area-based Pnmax, this indicates that shared LMA normalization alone did not account for the observed association in this dataset. The result represents a species-level association and does not establish a causal or independent biochemical effect of Narea, or a synergistic effect between Narea and SLA. The positive association between AQY and Narea should likewise be interpreted as an exploratory species-level association. Correlations calculated separately for trees and shrubs are presented only as exploratory results because of the small subgroup sample sizes (trees, n = 11; shrubs, n = 10). Because Pnmax, AQY, LSP, LCP, and Rd were derived from the same fitted light-response curve, correlations among these parameters were not interpreted as independent biological associations. Exploratory correlation matrices for tree and shrub species are provided in Figure S2. Raw *p* values and Benjamini–Hochberg FDR-adjusted q values for all 28 pre-specified trait–photosynthetic parameter correlations are provided in Table S3. In an auxiliary species-level regression, Rd was not significantly associated with Narea (slope = 0.2707, 95% CI = −0.1866 to 0.7281, *p* = 0.2305, R^2^ = 0.0747). This auxiliary analysis was not included in the pre-specified 28-correlation FDR family. The pooled species-level correlations are presented in Figure 3.

### 2.4. Species-Level Regression Models of Pnmax and AQY

Species-level candidate regression models were fitted using the mean values of the 21 sampled species (Table 2). For Pnmax, the Narea-only model had the lowest AICc (adjusted R^2^ = 0.440, AICc = 108.87, *p* < 0.001). Adding LA did not provide significant additional explanatory value (incremental F1,18 = 0.506, *p* = 0.486); the Narea + LA model had a higher AICc (ΔAICc = 2.51) and a lower leave-one-species-out cross-validated R^2^ (0.321 vs. 0.373). For AQY, the candidate models were statistically indistinguishable in this dataset. Although adjusted and cross-validated R^2^ were higher for the Narea + LA model, the AICc difference was small and the incremental F-test was not significant; therefore, the data do not establish that adding LA improves the model. Models combining Narea and SLA were excluded from primary interpretation because these variables are mathematically coupled through LMA. The regression results are interpreted as descriptive species-level associations rather than evidence of independent or synergistic trait effects. *Model coefficients and their 95% confidence intervals are reported in Table 3*.

## 3. Discussion

### 3.1. Differences in Photosynthetic Characteristics Among Plants with Different Growth Forms

The key parameters of the light-response curve, including Pnmax, AQY, LSP, and LCP, are commonly used to characterize plant photosynthetic performance under different light conditions [43]. Pnmax represents the maximum net photosynthetic capacity under saturating light, whereas AQY reflects the initial efficiency of carbon assimilation under low irradiance [44]. LSP and LCP describe the light intensity required for photosynthetic saturation and for a zero net carbon balance, respectively [45,46]. Previous studies have related variation in these parameters to leaf traits, resource-use strategies, and light environments [46,47,48].

In the present species-level analysis, no statistically significant differences between trees and shrubs remained for Pnmax, AQY, LSP, or LCP after false discovery rate correction. Although these parameters varied among the sampled species, the current data do not support a robust conclusion that shrubs have greater light-use adaptability, higher light-compensation capacity, or a competitive advantage under high-light conditions than trees. These comparisons should be interpreted cautiously because the numbers of tree and shrub species were limited and substantial variation existed within each growth form.

### 3.2. Associations Between Leaf Traits and Photosynthetic Characteristics

At the species level, the FDR-adjusted correlation analysis retained positive associations of Narea with both Pnmax and AQY. This pattern is broadly consistent with the expectation that leaf nitrogen investment is associated with photosynthetic capacity [27,49]. The mass-basis correlation between Pnmax and Nmass should not be interpreted as corroboration because both variables share LMA as a denominator. In contrast, LMA was not significantly correlated with area-based Pnmax or Narea, and a partial Spearman analysis controlling for LMA retained a positive Pnmax–Narea association (partial ρ = 0.762, *p* < 0.001). The partial association was stronger than the zero-order association (partial Spearman’s ρ = 0.762 vs. Spearman’s ρ = 0.673), a pattern consistent with LMA statistically masking rather than inflating the Pnmax–Narea association in this dataset. This interpretation is consistent with the opposite directions of the correlations of LMA with Narea (ρ = 0.187) and Pnmax (ρ = −0.265), although neither correlation was statistically significant. The association remains observational, however, and should not be interpreted as evidence of a causal or independent biochemical effect. Potential normalization artifacts in mass- and area-based trait comparisons have been discussed previously [40,41,42].

No associations between SLA or LA and the photosynthetic parameters remained significant after FDR correction in the overall species-level analysis. This absence of a retained SLA–area-based Pnmax association is not inconsistent with the global leaf economics spectrum, in which mass-based assimilation is strongly related to LMA, whereas area-based assimilation is generally only weakly related to LMA and therefore to its inverse, SLA [48]. Chen et al. [9] examined light-response curve parameters in 75 woody species and incorporated leaf habit and sun–shade contrasts, providing a broader trait-based framework than the present local study. In comparison, our analysis of 21 sampled species retained only the Narea–Pnmax and Narea–AQY associations after multiple-testing correction, highlighting the limited generality of trait-based relationships within this smaller secondary-forest dataset. Previous studies have shown that leaf structural traits are associated with plant resource-use strategies [38], whereas chlorophyll-related proxies for photosynthetic capacity have been evaluated in other systems [50]; however, such relationships were not retained in the present overall analysis after multiple-testing correction. The subgroup analyses of trees and shrubs were exploratory because each subgroup contained only 10 or 11 species. Therefore, the present data do not support strong conclusions about different trait-based photosynthetic strategies between trees and shrubs.

Among the limited prespecified candidate models, the Narea-only model had the lowest AICc for Pnmax. For AQY, the Narea-only and Narea + LA models had similar support (ΔAICc = 0.41), and the data did not clearly distinguish between them. The incremental F-tests did not show a significant improvement after adding LA for either Pnmax (*p* = 0.486) or AQY (*p* = 0.135). The models had moderate explanatory power and were not independently validated; they should therefore be interpreted as descriptive species-level models, not as transferable prediction equations.

### 3.3. Leaf Trait Variations Across Sampled Woody Species

The sampled woody species showed considerable variation in leaf structural, biochemical, and stoichiometric traits. Such variation is expected among co-occurring woody species because leaf traits reflect differences in leaf construction, biomass investment, nutrient status, and resource-use strategies [9,50]. However, the present dataset was not designed to establish fixed functional differences between trees and shrubs. Although some traits differed between growth forms in the descriptive comparisons, the numbers of tree and shrub species were limited, and trait variation within each growth form was substantial. Therefore, these differences should not be interpreted as evidence that one growth form consistently has greater resource-use efficiency, drought resistance, or photosynthetic adaptability than the other.

Several leaf traits were calculated from shared measurements. For example, SLA, LDMC, and area-based nutrient contents involve leaf area, dry mass, or fresh mass. Their covariation may therefore partly reflect shared calculation components. Further studies including more species, independent individuals, and repeated sampling across environmental conditions are needed to distinguish biological trait coordination from mathematical dependence among derived traits.

### 3.4. Study Scope and Limitations

This study has several limitations. First, the analyses were based on species means for 21 sampled woody species from three plots within one local secondary-forest region. The numbers of tree and shrub species were limited, and growth form was partly associated with leaf habit; therefore, growth-form comparisons and subgroup correlations should be regarded as exploratory. Repeated leaf-level measurements were averaged before species-level inference and were not treated as independent biological replicates. Second, the light-response curves were fitted from measurements obtained under standardized chamber conditions. Seventeen species had finite LSP estimates within the measured irradiance range, whereas two LSP estimates were extrapolated and two were undefined because β was estimated at zero. The LSP-related results should therefore be interpreted cautiously. In addition, Narea, SLA, and other area-based traits share mathematical components through LMA or leaf area. Although the partial Spearman analysis suggests that LMA normalization alone did not explain the Pnmax–Narea association in this dataset, the observational design cannot establish a causal mechanism. Future studies should include more species and independent individuals across multiple sites and microenvironments, together with directly measured mass- and area-based traits. Such work would help evaluate the generality of the observed associations and separate physiological relationships from mathematical coupling. Because planting status was not recorded, we cannot determine whether non-native or commonly cultivated taxa, including *Jacaranda mimosifolia*, *Bougainvillea glabra*, *Acer palmatum*, and *Michelia alba*, were planted or naturally established. The sampled assemblage should therefore be interpreted as the woody taxa present at the study sites, rather than as a representative sample of native secondary-forest vegetation. Mean height, DBH, and individual planting status were not systematically recorded during the original field survey and are therefore unavailable for retrospective reporting.

## 4. Materials and Methods

### 4.1. Study Sites

The coordinates of the Tongluo Mountain area are 29.8000° N, 106.7500° E and 29.7700° N, 106.7650° E; the approximate coordinates of the area near the Chongqing Bureau of Geology and Minerals Exploration are 29.6194° N, 106.5179° E. The study region is located within the subtropical humid evergreen broad-leaved forest zone based on a regional bioclimatic classification. The average annual temperature is 18.6 °C, and the average annual precipitation is approximately 1100 mm, with higher rainfall in summer and lower rainfall in winter. The sampled vegetation represented local secondary-forest vegetation. No artificial clipping or hedge management was observed for the sampled shrubs. The dominant soil types were red soil and yellow soil. Plot-level GPS coordinates and topographic measurements were not systematically recorded during the original field survey and therefore cannot be reported retrospectively.

### 4.2. Sample Collection and Analysis

Field preparation and leaf-trait sampling began in July 2023, whereas the light-response curves were measured from August to September 2023. Measurements were conducted during the same summer growing season using fully expanded and healthy leaves. Based on plant community ecology research methods, the vegetation growth forms were categorized into trees and shrubs. Three 100 × 100 m plots were established within the study area, each subdivided into 20 × 20 m quadrats for community surveys. Based on the plot survey results, field sampling feasibility, and the common occurrence of species in the study area, 21 common and representative woody species were selected for this experiment, including 11 tree species and 10 shrub species. Leaf-habit categories used in the primary analysis are reported in Table 4. Because the factorial models required a binary leaf-habit factor, semi-evergreen taxa were assigned to the evergreen or deciduous category specified in the Table 4 note. It should be noted that the main objective of this study was to analyze the relationships between leaf traits and photosynthetic capacity. The preliminary plot survey was mainly used to identify common woody species in the study area, and complete quantitative measurements of importance values, relative coverage, or relative abundance for all community species were not conducted. Therefore, the selected species can represent the trait variation in common woody plants in the study area, but they cannot fully represent the entire species composition of the local plant community (see Table 4 for detailed species information). For each species, two healthy individuals with similar growth vigor and no visible pest or disease damage were selected within the same plot. From each selected individual, three fully expanded, south-facing leaves of similar size were selected from outer-crown or relatively high-light positions on 1–2-year-old branches. These selection criteria were used to reduce variation in within-canopy light conditions. The selected leaves were used for light-response curve measurements. After gas-exchange measurements, the same leaves were collected to determine leaf dry matter content (LDMC), specific leaf area (SLA), leaf area (LA), SPAD value, carbon content per unit leaf area (Carea), nitrogen content per unit leaf area (Narea), and carbon-to-nitrogen ratio (C/N).

### 4.3. Measurement of Light Response Curves

Light response curves were measured from August to September 2023 on sunny, windless days. All measurements were conducted between 8:30 and 11:30 a.m. to avoid midday photosynthetic depression. For each species, two individual plants were sampled from the same plot. For each plant, three healthy, fully expanded, similar-sized, south-facing leaves from outer-crown or relatively high-light positions on 1- to 2-year-old branches were selected to minimize variation in within-canopy light conditions. Measurements were performed using the LI-6800 Portable Photosynthesis System (LI-COR Biosciences, Lincoln, NE, USA). Repeated leaf-level measurements were averaged before the species-level analysis and were not treated as independent biological replicates. Prior to data collection, leaves were acclimated in the chamber for approximately 30 min under the stated measurement conditions to achieve stable gas exchange. The reference CO_2_ concentration was maintained at 400 µmol mol^−1^. The nominal light levels were 0, 30, 70, 100, 150, 200, 300, 600, 900, 1200, and 1500 μmol m^−2^ s^−1^; an additional level of 1800 μmol m^−2^ s^−1^ was recorded in some runs. Accordingly, the original LI-6800 records contained 11 or 12 measured irradiance levels per run. The leaf chamber temperature (Tblock) was set at 25 °C, and relative humidity (RH) was controlled at 65 ± 5%. For leaves smaller than the chamber aperture, the projected leaf area enclosed within the chamber was measured using Fiji (ImageJ 2.18.0), and the actual enclosed leaf area was used to correct gas-exchange calculations in the LI-6800 system. Because gas exchange was measured under controlled chamber conditions (25 °C, 65 ± 5% RH, and 400 µmol mol^−1^ CO_2_), the reported photosynthetic parameters represent standardized measurement values rather than instantaneous in situ values. At each light level, data were recorded only after net photosynthetic rate and stomatal conductance had reached stable values. Figure S1 illustrates a typical photosynthetic light-response curve and defines the key parameters used to evaluate photosynthetic capacity in this study, including the maximum net photosynthetic rate Pnmax, light saturation point LSP, and light compensation point LCP. The results were consequently interpreted as standardized chamber measurements under the controlled conditions described above.

The original LI-6800 files were available, but could not be reliably linked to the species, leaf samples, and measurement runs included in the analysis. Consequently, flow rate, fan speed, leaf temperature, leaf-to-air VPD, and the stabilization time at each light step could not be verified retrospectively. The pre-measurement acclimation irradiance and duration could not be reliably established from the available records. These values are therefore not reported.

### 4.4. Measurement of Leaf Traits

For each sampled species, the same leaves used for the light-response curve measurements were also used to determine SPAD value, LA, SLA, LDMC, Carea, Narea, and C/N ratio. SPAD values were measured using a SPAD-502 m (Konica Minolta, Tokyo, Japan). Three readings were obtained per leaf and averaged. LA was determined by scanning the leaves on A4 paper and calculating their actual area using Fiji (ImageJ) software. The area of a single leaf was determined by dividing the total scanned area by the number of leaves. Leaf fresh weight was measured using a microbalance, after which the selected leaves were submerged in water for 12 h in the dark to reach saturation. After removing excess surface moisture with absorbent paper, the saturated fresh weight (SFW) was recorded. The leaves were then oven-dried at 105 °C for 15 min to inactivate enzymes, followed by drying at 80 °C until a constant weight was achieved and the dry weight (DW) was recorded. SLA was calculated as LA/DW and LDMC as DW/SFW. Finally, dried leaves were ground into a fine powder using a mortar, and their carbon and nitrogen contents were analysed using a vario MAX cube elemental analyser (Elementar Analysensysteme GmbH, Langenselbold, Germany).

In this study, the measured variables were classified into three categories according to their ecological and physiological meanings. The first category comprised photosynthetic physiological parameters, including the maximum net photosynthetic rate (Pnmax), apparent quantum yield (AQY), light saturation point (LSP), and light compensation point (LCP), all of which were derived from the fitted light-response curves and used to characterize leaf photosynthetic capacity and light-acclimation characteristics. The second category comprised leaf structural traits, including specific leaf area (SLA), leaf area (LA), and leaf dry matter content (LDMC), which mainly reflect leaf morphological construction, resource-acquisition strategies, and structural investment. The third category comprised leaf biochemical or stoichiometric traits, including SPAD value, carbon content per unit leaf area (Carea), nitrogen content per unit leaf area (Narea), and the carbon-to-nitrogen ratio (C/N), which reflect leaf pigment status, carbon and nitrogen allocation, and nutrient investment. Although Narea is not a physiological trait in the strict sense, it reflects nitrogen investment per unit leaf area in photosynthesis-related proteins and enzymes, such as Rubisco, and is therefore closely associated with area-based photosynthetic capacity. Thus, Narea was included as a biochemical indicator in the exploratory species-level analyses of variation in Pnmax and other photosynthetic parameters. Leaf nitrogen content was first measured on a mass basis and then converted to nitrogen content per unit leaf area (Narea). Specifically, leaf mass per area (LMA) was calculated as the ratio of leaf dry mass to leaf area, and LMA is the inverse of SLA. Narea was calculated as the product of mass-based nitrogen content (Nmass) and LMA, namely Narea = Nmass × LMA. Therefore, Narea represents nitrogen investment per unit leaf area and can be used to characterize the biochemical basis of photosynthesis at the leaf-area scale. Because Narea is calculated as Nmass × LMA and LMA is the inverse of SLA, Narea and SLA are mathematically coupled. Models combining Narea and SLA were therefore not used as primary mechanistic models, and associations involving these variables were interpreted cautiously as descriptive species-level relationships.

### 4.5. Data Analysis

For each species, repeated leaf-level measurements were first averaged at each nominal irradiance level. A single species-level Pn–I curve, containing 11 or 12 irradiance levels depending on whether the 1800 μmol m^−2^ s^−1^ level was recorded, was then fitted using the modified rectangular hyperbola equation described by Ye [51,52]:

$$P_{n}\left(I\right)=\alpha \frac{\left(1-\mathrm{\beta I}\right)}{1+\mathrm{\gamma I}}I-R_{d}$$

where Pn(I) is the net photosynthetic rate at a given photosynthetic photon flux density (I); I is photosynthetically active radiation (PAR); α is the initial slope of the fitted light-response curve at low irradiance; β is an empirical coefficient describing photoinhibition or dynamic down-regulation at high irradiance; γ is an empirical coefficient related to light saturation; and Rd is the dark respiration rate. At I = 0, the model gives Pn = −Rd.

The parameters α, β, γ, and Rd were estimated by nonlinear least-squares fitting of the observed Pn − I data. AQY was estimated separately as the slope of an ordinary least-squares regression fitted to LI-6800 observations recorded at the four nominal low-light settings (0, 30, 70, and 100 μmol m^−2^ s^−1^), multiplied by 1000. No interpolated Pn values were used, and AQY was not treated as identical to the fitted α parameter. The light saturation point (LSP) was calculated as the irradiance at which the fitted curve reached its maximum value:

$$\mathrm{LSP}=\frac{\sqrt{1+\frac{\gamma }{\beta }}-1}{\gamma }$$

LSP estimates were classified according to their fitting status. Estimates were considered accepted when they were finite and within the measured irradiance range (≤1800 μmol m^−2^ s^−1^). Estimates exceeding 1800 μmol m^−2^ s^−1^ were classified as extrapolated, whereas LSP was classified as undefined when β was estimated at its boundary value of zero. For fits with β > 0, Pnmax was calculated by evaluating the fitted curve at the fitted LSP. Accordingly, Pnmax estimates for *Jacaranda mimosifolia* and *Vitex negundo* var. *cannabifolia* were classified as extrapolated. For *Osmanthus fragrans* and *Rhododendron simsii*, β = 0, so neither a finite LSP nor a finite Pnmax exists. For these two species, the asymptotic net photosynthetic rate, calculated as α/γ − Rd, was reported separately (3.33 and 4.29 μmol m^−2^ s^−1^, respectively) and was not interpreted as Pnmax. Parameter estimates, approximate 95% confidence intervals, fit R^2^ values, and LSP fitting status are reported in Table 1. The light compensation point (LCP) was calculated as the irradiance at which the fitted net photosynthetic rate equaled zero, i.e., Pn(I) = 0. The fitted curves and parameter estimates were visually checked against the observed data.

All inferential analyses used species means as independent observations (*n* = 21). Differences in photosynthetic parameters and leaf traits were evaluated using species-level linear models including growth form (tree or shrub), leaf habit (evergreen or deciduous), and their interaction. Results for trees (*n* = 11) and shrubs (*n* = 10) were interpreted cautiously because of the limited subgroup sample sizes. Spearman correlation analyses were conducted between photosynthetic parameters and leaf traits using species means. The 28 trait–photosynthetic parameter correlations in the pooled species-level analysis were treated as one family of tests, and their *p* values were adjusted using the Benjamini–Hochberg false discovery rate procedure. For the growth-form and leaf-habit models, the *p* values for the three terms (growth form, leaf habit, and their interaction) across the 11 response variables were adjusted together using the Benjamini–Hochberg procedure. Species-level linear regression models were used to evaluate associations between Pnmax or AQY and Narea, SLA, and LA. Candidate predictors (Narea, SLA, and LA) were specified a priori based on the original study objectives and their established relevance to leaf structure and nutrient investment; no stepwise or all-subsets selection procedure was used. Candidate models were compared using adjusted R^2^, the small-sample corrected Akaike information criterion (AICc), and leave-one-species-out cross-validation. Because Narea and SLA are mathematically coupled through LMA, models combining Narea and SLA were not used as primary mechanistic models. Structural equation modeling was not used because the species-level sample size was insufficient for reliable estimation of the previously specified SEM. All results were interpreted as exploratory interspecific associations rather than causal effects. Statistical analyses were conducted using IBM SPSS Statistics 29.0 (IBM Corp., Armonk, NY, USA). Figures were prepared using OriginPro 2022 (OriginLab Corporation, Northampton, MA, USA). Leaf area was measured using Fiji (ImageJ 2.18.0) [53]. Normality was assessed using the Shapiro–Wilk test and Q–Q plots, and homogeneity of variance was assessed using Levene’s test. Shapiro–Wilk tests indicated non-normal residuals for AQY (W = 0.873, *p* = 0.011), LSP (W = 0.700, *p* < 0.001), and LCP (W = 0.841, *p* = 0.003), whereas Levene’s tests indicated unequal variances for LSP (F = 4.420, *p* = 0.020), LCP (F = 3.801, *p* = 0.030), and log_10_-transformed LA (F = 12.999, *p* < 0.001); tests for the other responses were nonsignificant (*p* > 0.05). Because LA showed a right-skewed distribution, its log10-transformed values were used in parametric analyses. Spearman correlations were performed on the original values because they are rank-based and do not require normally distributed variables. Additional pre-specified diagnostic Spearman correlations were conducted to compare area- and mass-based relationships and to evaluate potential LMA-related normalization effects; these diagnostic results are reported in Table S1.

## 5. Conclusions

This study quantified photosynthetic parameters and leaf traits in 21 common woody species from subtropical secondary-forest vegetation in Chongqing. At the species level, Narea was positively associated with both Pnmax and AQY after false discovery rate correction. No robust overall differences in Pnmax, AQY, LSP, or LCP remained between trees and shrubs after accounting for multiple testing. The smallest growth form × leaf habit group contained only four species, so simple tree–shrub contrasts should also be interpreted cautiously. Among the prespecified candidate models, the Narea-only model had the lowest AICc for Pnmax. For AQY, the Narea-only and Narea + LA models had similar support (ΔAICc = 0.41), and the data did not clearly distinguish between them. Adding LA did not significantly improve either model in the incremental F-tests. The positive Pnmax–Narea association remained after controlling for LMA, indicating that shared LMA normalization alone did not explain the association in this dataset. Nevertheless, this observational association does not establish causation. The limited number of species and the absence of an independent validation dataset restrict broader inference. Future studies should include more species, independent replication across sites and microenvironments, and directly measured mass- and area-based traits to separate physiological relationships from mathematical coupling.

## Figures and Tables

**Table 1:** Parameters and goodness of fit of modified rectangular hyperbola models for the 21 species-level light-response curves.

**Species No.**	**Scientific Name**	**α (95% CI)**	**β (95% CI)**	**γ (95% CI)**	**Rd (95% CI)**	**Fit R** **^2^**	**LSP Status**	**LSP/Pnmax Status**
1	*Ficus concinna*	0.104925 (0.068699, 0.141151)	0.000263 (0.000204, 0.000322)	0.015498 (0.009536, 0.021460)	0.6950 (0.2041, 1.1859)	0.9844	Accepted	Finite (LSP within measured range)
2	*Osmanthus fragrans*	0.058134 (0.039266, 0.077001)	0 (boundary; CI not estimable)	0.016007 (0.010143, 0.021872)	0.3010 (0.0529, 0.5491)	0.9925	β = 0; LSP undefined	Undefined (β = 0; no finite LSP or Pnmax)
3	*Elaeocarpus decipiens*	0.086971 (0.063955, 0.109987)	0.000088 (0.000009, 0.000168)	0.005018 (0.003162, 0.006874)	1.0618 (0.2865, 1.8371)	0.9951	Accepted	Finite (LSP within measured range)
4	*Cinnamomum camphora*	0.084189 (0.065394, 0.102985)	0.000144 (0.000080, 0.000208)	0.004236 (0.002865, 0.005607)	0.8467 (0.1358, 1.5576)	0.996	Accepted	Finite (LSP within measured range)
5	*Michelia alba*	0.085820 (0.067488, 0.104151)	0.000166 (0.000114, 0.000217)	0.006613 (0.004793, 0.008432)	0.7263 (0.2211, 1.2315)	0.9956	Accepted	Finite (LSP within measured range)
6	*Cinnamomum japonicum*	0.109933 (0.057199, 0.162666)	0.000141 (0.000049, 0.000232)	0.016578 (0.007712, 0.025445)	0.3683 (−0.3032, 1.0398)	0.9764	Accepted	Finite (LSP within measured range)
7	*Ficus virens*	0.097897 (0.067639, 0.128154)	0.000154 (0.000089, 0.000218)	0.011530 (0.007376, 0.015684)	0.4839 (−0.0483, 1.0161)	0.9901	Accepted	Finite (LSP within measured range)
8	*Cerasus serrulata*	0.100458 (0.073089, 0.127826)	0.000193 (0.000135, 0.000250)	0.009398 (0.006327, 0.012469)	1.0415 (0.4698, 1.6132)	0.9917	Accepted	Finite (LSP within measured range)
9	*Acer palmatum*	0.094633 (0.068871, 0.120395)	0.000196 (0.000150, 0.000242)	0.021054 (0.014816, 0.027292)	0.3580 (0.0965, 0.6195)	0.9913	Accepted	Finite (LSP within measured range)
10	*Jacaranda mimosifolia*	0.060879 (0.046468, 0.075290)	0.000013 (−0.000083, 0.000109)	0.003221 (0.001964, 0.004479)	1.9826 (1.3346, 2.6306)	0.9974	Extrapolated	Extrapolated (LSP and Pnmax)
11	*Koelreuteria bipinnata*	0.058212 (0.025575, 0.090848)	0.000252 (0.000120, 0.000384)	0.004086 (0.000812, 0.007360)	1.6350 (0.3713, 2.8987)	0.9647	Accepted	Finite (LSP within measured range)
12	*Pyracantha fortuneana*	0.058753 (0.051267, 0.066239)	0.000319 (0.000275, 0.000362)	0.002263 (0.001712, 0.002814)	1.7606 (1.3742, 2.1470)	0.9985	Accepted	Finite (LSP within measured range)
13	*Photinia serrulata*	0.076408 (0.058278, 0.094539)	0.000194 (0.000105, 0.000284)	0.003780 (0.002324, 0.005236)	1.1123 (0.4138, 1.8108)	0.996	Accepted	Finite (LSP within measured range)
14	*Bougainvillea glabra*	0.071831 (0.059394, 0.084268)	0.000205 (0.000148, 0.000261)	0.005549 (0.004172, 0.006926)	0.7464 (0.3759, 1.1169)	0.9976	Accepted	Finite (LSP within measured range)
15	*Viburnum odoratissimum*	0.080351 (0.065515, 0.095187)	0.000126 (0.000055, 0.000197)	0.004950 (0.003574, 0.006326)	1.2274 (0.7488, 1.7060)	0.9979	Accepted	Finite (LSP within measured range)
16	*Pittosporum tobira*	0.064010 (0.046023, 0.081996)	0.000136 (0.000040, 0.000231)	0.006512 (0.003974, 0.009050)	0.6074 (0.1302, 1.0846)	0.9946	Accepted	Finite (LSP within measured range)
17	*Rhododendron simsii*	0.019700 (0.012488, 0.026912)	0 (boundary; CI not estimable)	0.003619 (0.001317, 0.005920)	1.1561 (0.8705, 1.4417)	0.9946	β = 0; LSP undefined	Undefined (β = 0; no finite LSP or Pnmax)
18	*Ligustrum quihoui*	0.097042 (0.062369, 0.131716)	0.000042 (−0.000063, 0.000146)	0.013667 (0.007830, 0.019504)	0.4369 (−0.0684, 0.9422)	0.992	Accepted	Finite (LSP within measured range)
19	*Vitex negundo* var. *cannabifolia*	0.077551 (0.059181, 0.095921)	0.000064 (−0.000053, 0.000180)	0.003410 (0.001997, 0.004823)	1.0720 (0.3179, 1.8261)	0.9973	Extrapolated	Extrapolated (LSP and Pnmax)
20	*Viburnum chinshanense*	0.068480 (0.055402, 0.081559)	0.000183 (0.000103, 0.000263)	0.002996 (0.001977, 0.004015)	1.2665 (0.6869, 1.8461)	0.9976	Accepted	Finite (LSP within measured range)
21	*Coriaria nepalensis*	0.093181 (0.063140, 0.123223)	0.000051 (−0.000054, 0.000156)	0.009994 (0.005915, 0.014072)	1.3128 (0.7425, 1.8831)	0.9937	Accepted	Finite (LSP within measured range)

Notes: Parameters were estimated from species-level mean light-response curves. Values in parentheses indicate 95% confidence intervals. AQY was estimated separately from the linear slope at low irradiance (0–100 μmol m^−2^ s^−1^) and is therefore not identical to the fitted α parameter. “Accepted” indicates that LSP was finite and within the observed irradiance range; “Extrapolated” indicates that the fitted LSP exceeded 1800 μmol m^−2^ s^−1^; β = 0 indicates a boundary estimate for which LSP was undefined. Pnmax status follows the status of the corresponding LSP. For species with β = 0, no finite Pnmax exists; the asymptotic net photosynthetic rate is reported separately in the text.

**Table 2:** Comparison of species-level candidate regression models for *Pn*max and AQY across 21 woody species. ΔAICc is calculated relative to the lowest-AICc model for each response. LOOCV R^2^, leave-one-species-out cross-validated coefficient of determination. *Pn*max, maximum net photosynthetic rate; AQY, apparent quantum yield; Narea, nitrogen content per unit leaf area; SLA, specific leaf area; LA, leaf area. Narea + LA indicates an additive two-predictor model.

Response (Unit)	Model	*n*	Adjusted R^2^	AICc	ΔAICc	LOOCV R^2^	Model *p* Value
*P*_nmax_ (μmol m^−2^ s^−1^)	Narea	21	0.440	108.87	0	0.373	<0.001
	SLA	21	0.085	119.18	10.31	−0.076	0.107
	LA	21	−0.052	122.10	13.23	−0.167	0.899
	Narea + LA	21	0.425	111.37	2.51	0.321	0.003
AQY (mmol mol^−1^)	Narea	21	0.308	150.85	0	0.226	0.005
	SLA	21	−0.012	158.85	8	−0.118	0.395
	LA	21	0.080	156.84	5.98	−0.052	0.114
	Narea + LA	21	0.357	151.26	0.41	0.269	0.007

**Table 3:** Estimated coefficients and 95% confidence intervals for species-level candidate regression models across 21 woody species. *Pn*max, maximum net photosynthetic rate; AQY, apparent quantum yield; Narea, nitrogen content per unit leaf area; SLA, specific leaf area; LA, leaf area. Narea + LA indicates an additive two-predictor model.

Response	Model	Term	Estimate	95% CI Lower	95% CI Upper
*P_nmax_*	Narea	Intercept	0.8275	−3.3263	4.9813
	Narea	Narea	5.4478	2.6581	8.2375
	SLA	Intercept	5.9989	2.4486	9.5492
	SLA	SLA	0.0185	−0.0044	0.0415
	LA	Intercept	8.6565	5.9466	11.3663
	LA	LA	−0.0046	−0.0799	0.0707
	Narea + LA	Intercept	1.1644	−3.1760	5.5049
	Narea + LA	Narea	5.5754	2.7131	8.4377
	Narea + LA	LA	−0.0191	−0.0755	0.0373
AQY (mmol mol^−1^)	Narea	Intercept	25.7574	14.4699	37.0449
	Narea	Narea	11.4036	3.8229	18.9843
	SLA	Intercept	38.5299	29.3991	47.6607
	SLA	SLA	0.0245	−0.0345	0.0836
	LA	Intercept	38.1884	31.9918	44.385
	LA	LA	0.1363	−0.0359	0.3085
	Narea + LA	Intercept	23.8403	12.6206	35.0599
	Narea + LA	Narea	10.6775	3.2788	18.0763
	Narea + LA	LA	0.1086	−0.0371	0.2544

All models were fitted using species means (*n* = 21). Estimate indicates the fitted regression coefficient. CI, confidence interval. Pnmax, maximum net photosynthetic rate; AQY, apparent quantum yield; Narea, nitrogen content per unit leaf area; SLA, specific leaf area; LA, leaf area.

**Table 4:** List of 21 Common Plant Species.

Order	Species	Family	Growth Form	Leaf Habit
1	*Ficus concinna* (Miq.) Miq.	Moraceae	tree	evergreen
2	*Osmanthus fragrans* (Thunb.) Lour.	Oleaceae	tree	evergreen
3	*Elaeocarpus decipiens* Hemsl.	Elaeocarpaceae	tree	evergreen
4	*Cinnamomum camphora* (L.) presl	Lauraceae	tree	evergreen
5	*Michelia alba* DC.	Magnoliaceae	tree	evergreen
6	*Cinnamomum japonicum* Sieb.	Lauraceae	tree	evergreen
7	*Ficus virens Aiton*	Moraceae	tree	deciduous
8	*Cerasus serrulata* (Lindl.) G.	Rosaceae	tree	deciduous
9	*Acer palmatum* Thunb.	Sapindaceae	tree	deciduous
10	*Jacaranda mimosifolia* D. Don	Bignoniaceae	tree	deciduous
11	*Koelreuteria bipinnata* Franch.	Sapindaceae	tree	deciduous
12	*Pyracantha fortuneana* (Maxim.)	Rosaceae	shrub	evergreen
13	*Photinia serrulata* Lindl.	Rosaceae	shrub	evergreen
14	*Bougainvillea glabra* Choisy.	Nyctaginaceae	shrub	evergreen
15	*Viburnum odoratissimum* Ker-Gawl.	Adoxaceae	shrub	evergreen
16	*Pittosporum tobira* (Thunb.) Ait.	Pittosporaceae	shrub	evergreen
17	*Rhododendron simsii* Planch.	Ericaceae	shrub	evergreen
18	*Ligustrum quihoui* Carr.	Oleaceae	shrub	deciduous
19	*Vitex negundo* L. *var. cannabifolia* (Sieb. et Zucc.) Hand.-Mazz.	Lamiaceae	shrub	deciduous
20	*Viburnum chinshanense* Graebn.	Adoxaceae	shrub	deciduous
21	*Coriaria nepalensis* Wall.	Coriariaceae	shrub	deciduous

Notes: Scientific names and authorities are retained as recorded in the original field dataset and have not been fully standardized against POWO. The leaf-habit column shows the binary categories used in the primary analysis. Ligustrum quihoui and Rhododendron simsii are treated as semi-evergreen taxa; for the primary analysis, L. quihoui was coded as deciduous and R. simsii as evergreen.

## Data Availability

The data supporting the findings of this study are not publicly available because they form part of an ongoing research project, but are available from the corresponding author upon reasonable request.

## Supplementary Materials

The following supporting information can be downloaded at: https://www.mdpi.com/article/10.3390/plants15213295/s1, Figure S1, representative fitted light-response curves; Figure S2, exploratory correlation matrices for tree and shrub species; Table S1, area- and mass-basis diagnostic analyses; Table S2, diagnostic comparison between empirical low-light AQY and the fitted α parameter; Table S3, Spearman correlations between leaf traits and photosynthetic parameters; and Table S4, complete species-level growth form, leaf habit, and interaction model results.

## Abbreviations

Abbreviations used in this manuscript.

Abbreviation	Definition
Pnmax	Maximum net photosynthetic rate
AQY	Apparent quantum yield
LSP	Light saturation point
LCP	Light compensation point
SLA	Specific leaf area
LDMC	Leaf dry matter content
LA	Leaf area
SPAD	SPAD meter value
Carea	Carbon content per unit leaf area
Narea	Nitrogen content per unit leaf area
C/N	Carbon-to-nitrogen ratio
LES	Leaf economics spectrum
