# Supplementary material for: Species-Level Associations of Leaf Traits and Photosynthetic Capacity in Common Woody Plants of Subtropical Secondary Forest Vegetation
Source: Plants (Basel). 2026 Oct 25;15(21):3295. doi: 10.3390/plants15213295 (PMC13765751; doi:10.3390/plants15213295)
Supplement: Supplementary file 1 [file plants-15-03295-s001.zip › plants-4447941-supplementary.pdf]

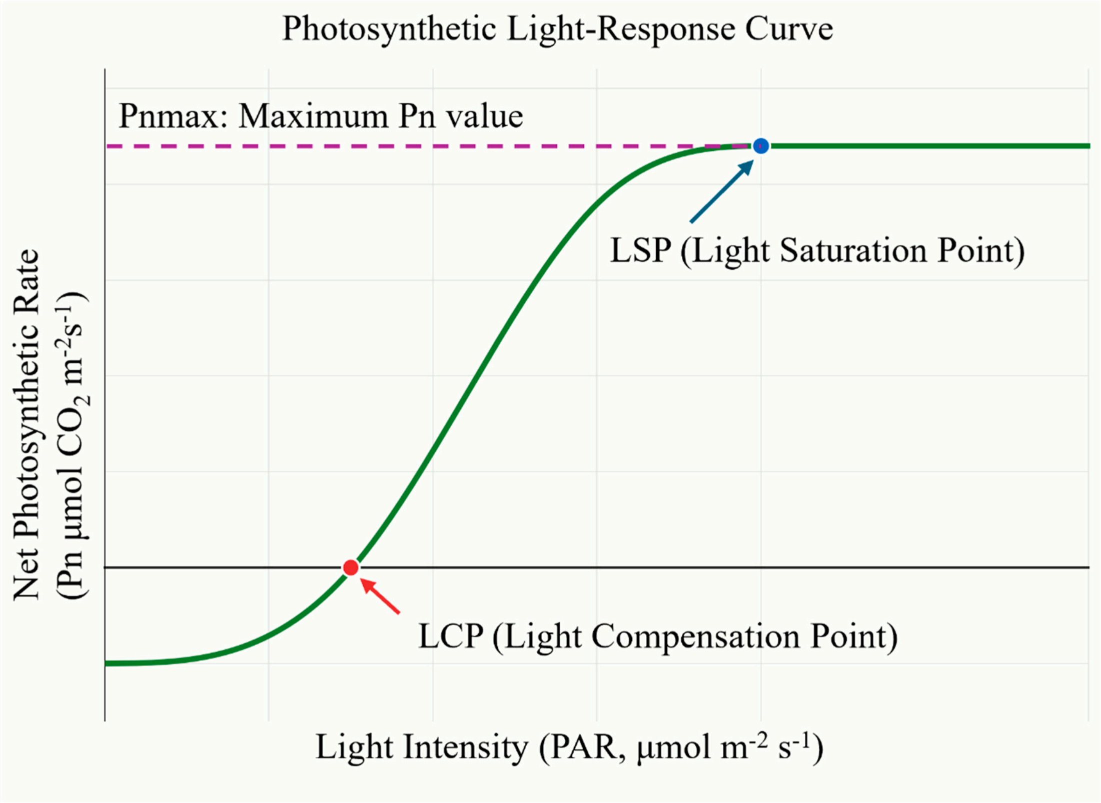

Figure S1. Photosynthetic light-response curve. Pnmax, maximum net photosynthetic rate; LSP, light saturation point; LCP, light compensation point; PAR, photosynthetically active radiation; CO_2, carbon dioxide.

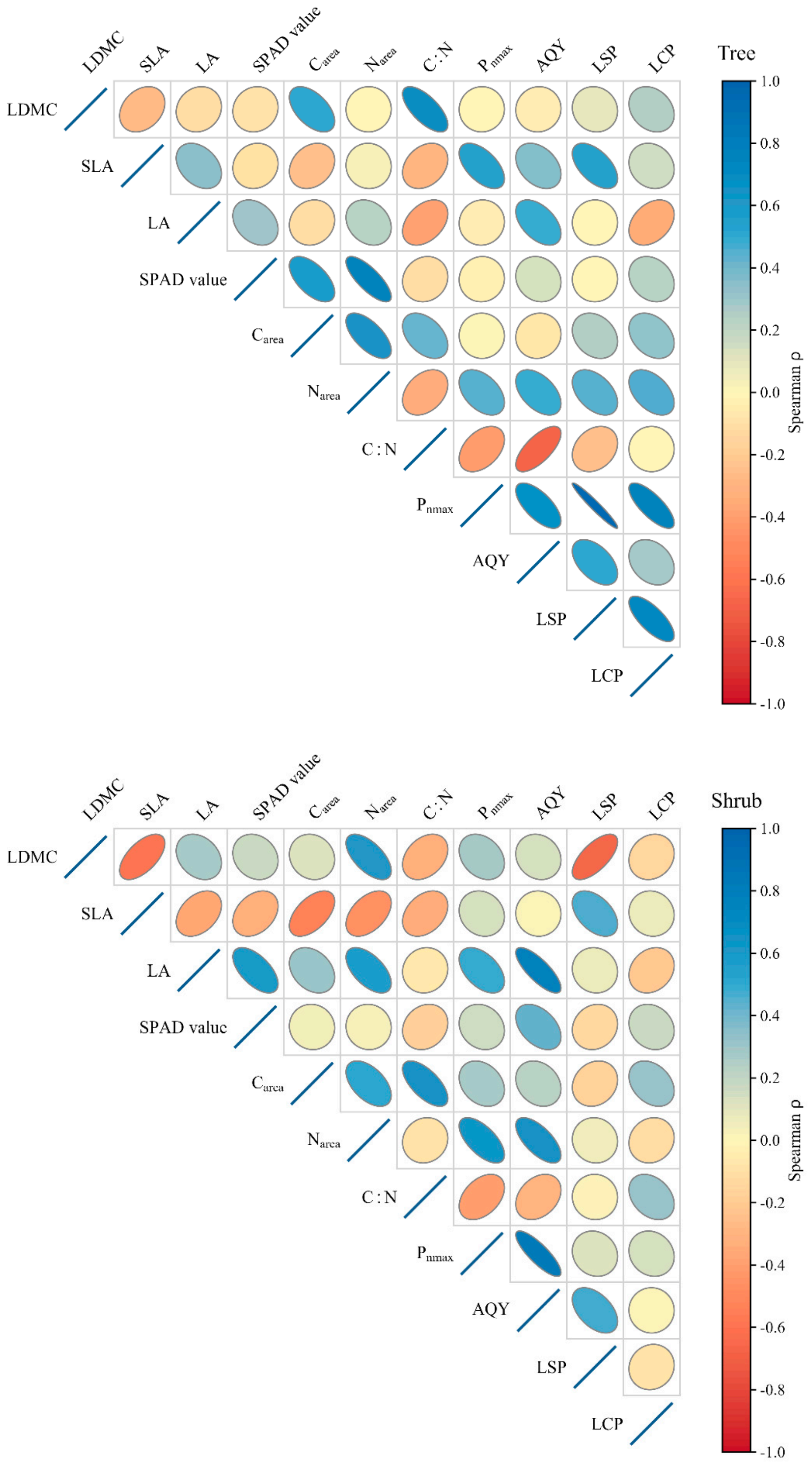

Figure S2. Exploratory Spearman correlation matrices between leaf traits and photosynthetic parameters for tree species (n = 11; upper panel) and shrub species (n = 10; lower panel), based on species means.

Ellipses represent Spearman's rho: blue and red indicate positive and negative correlations, respectively, and narrower ellipses indicate stronger correlations. No significance symbols are shown because these subgroup analyses are exploratory and have limited sample sizes. For correlations involving LSP, the effective sample sizes were n = 10 for trees and n = 9 for shrubs because one species in each group had no finite LSP estimate. Abbreviations are as defined in Figure 3.

Table S1. Area- and mass-basis diagnostic analyses of the relationships between leaf nitrogen and photosynthetic capacity across 21 woody species.

Diagnostic relationship	n	Spearman rho	Raw P	VIF	Interpretation
Pnmax (area basis) vs Narea	21	0.673	$P < 0.001$		Area-based association retained in the main analysis.
AQY (I = 0-100 μmol m^{-2} s^{-1}) vs Narea	21	0.666	$P < 0.001$		Low-light AQY association retained in the main analysis.
Pnmax (mass basis) vs Nmass	21	0.921	$P < 0.001$		The mass-basis correlation may be amplified by the shared LMA denominator and is not treated as independent corroboration.
Partial Spearman: Pnmax (area basis) vs Narea, controlling for LMA	21	0.762	P < 0.001		Positive association remains after controlling for LMA.
LMA vs Narea	21	0.187	$P = 0.417$		Checks whether variation in Narea is driven by LMA.
LMA vs Pnmax (area basis)	21	-0.265	$P = 0.246$		Checks whether variation in Pnmax is driven by LMA.
Narea vs SLA	21	-0.186	$P = 0.420$		Documents the correlation between the mathematically coupled variables.
VIF: Narea + LA model	21			1.018	VIF for Narea in the two-predictor Narea + LA model.

Notes: Pnmax (mass basis) was calculated as Pnmax (area basis)/LMA, and Nmass was calculated as Narea/LMA. VIF was calculated for the Narea + LA model. Partial Spearman correlation was calculated on

ranked variables, controlling for the rank of LMA.

Table S2. Diagnostic comparison between empirical low-light AQY and the fitted α parameter for the 21 species-level light-response curves.

Species No.	Scientific name	AQY (0–100; mmol mol⁻¹)	Low-light fit R^2	Fitted α (mol mol⁻¹)	α × 1000 / AQY
1	*Ficus concinna*	40.82	0.982	0.1049	2.57
2	*Osmanthus fragrans*	22.23	0.952	0.0581	2.62
3	*Elaeocarpus decipiens*	51.53	0.996	0.087	1.69
4	*Cinnamomum camphora*	51.62	0.999	0.0842	1.63
5	*Michelia alba*	47.67	0.992	0.0858	1.8
6	*Cinnamomum japonicum*	41.65	0.986	0.1099	2.64
7	*Ficus virens*	44.58	0.992	0.0979	2.2
8	*Cerasus serrulata*	48.49	0.992	0.1005	2.07
9	*Acer palmatum*	30.85	0.95	0.0946	3.07
10	*Jacaranda mimosifolia*	41.32	0.975	0.0609	1.47
11	*Koelreuteria bipinnata*	35.33	0.978	0.0582	1.65
12	*Pyracantha fortuneana*	42.93	0.997	0.0588	1.37
13	*Photinia serrulata*	49.12	0.998	0.0764	1.56
14	*Bougainvillea glabra*	43.3	0.988	0.0718	1.66
15	*Viburnum odoratissimum*	49.55	0.994	0.0804	1.62
16	*Pittosporum tobira*	35.8	0.998	0.064	1.79
17	*Rhododendron simsii*	17.1	0.971	0.0197	1.15
18	*Ligustrum quihoui*	41.69	0.988	0.097	2.33
19	*Vitex negundo* var. *cannabifolia*	52.17	0.998	0.0776	1.49
20	*Viburnum chinshanense*	47.27	0.999	0.0685	1.45
21	*Coriaria nepalensis*	44.52	0.988	0.0932	2.09

Notes: AQY (0–100) was estimated as the ordinary least-squares slope

of net photosynthetic rate against irradiance at nominal irradiance levels of 0, 30, 70, and 100 μmol m^{-}◎s^{-1}, multiplied by 1000, and is expressed in mmol mol^{-1}. Fitted α was estimated from the modified rectangular hyperbola equation and is expressed in mol mol^{-1}. The final column is the dimensionless ratio of fitted $\alpha \times 1000$ to empirical AQY.

Table S3. Spearman correlations between leaf traits and photosynthetic parameters across the sampled woody species.

Trait	Response	n	Spearman rho	Raw P	BH-FDR q
SPAD value	Pnmax	21	0.073	0.7541	0.8121
SPAD value	AQY	21	0.247	0.2809	0.6552
SPAD value	LSP	19	0.128	0.6013	0.7015
SPAD value	LCP	21	0.169	0.4644	0.6552
LDMC	Pnmax	21	0.129	0.5786	0.7015
LDMC	AQY	21	0.013	0.9554	0.9554
LDMC	LSP	19	−0.211	0.387	0.6552
LDMC	LCP	21	0.179	0.437	0.6552
SLA	Pnmax	21	0.282	0.2159	0.5494
SLA	AQY	21	0.155	0.5036	0.6714
SLA	LSP	19	0.437	0.0615	0.2553
SLA	LCP	21	0.129	0.5786	0.7015
LA	Pnmax	21	0.013	0.9554	0.9554
LA	AQY	21	0.474	0.0299	0.2096
LA	LSP	19	−0.228	0.3477	0.6552
LA	LCP	21	−0.379	0.09	0.315
Carea	Pnmax	21	0.195	0.3974	0.6552
Carea	AQY	21	0.092	0.691	0.7739
Carea	LSP	19	0.189	0.4372	0.6552
Carea	LCP	21	0.355	0.1148	0.3572
Narea	Pnmax	21	0.673	0.0008	0.0137
Narea	AQY	21	0.666	0.001	0.0137
Narea	LSP	19	0.433	0.0638	0.2553
Narea	LCP	21	0.283	0.2137	0.5494
C:N	Pnmax	21	−0.414	0.0619	0.2553
C:N	AQY	21	−0.549	0.0099	0.0924

C:N	LSP	19	−0.177	0.468	0.6552
C:N	LCP	21	0.171	0.4575	0.6552

Notes: P values were adjusted using the Benjamini–Hochberg false discovery rate procedure across all 28 trait–photosynthetic parameter correlations. Two species without finite LSP estimates were excluded from correlations involving LSP. After FDR correction, the positive associations of Narea with Pnmax and AQY remained significant (q = 0.0137 for both).

Table S4. Species-level linear models testing growth form, leaf habit, and their interaction for photosynthetic parameters and leaf traits.

Response	Term	n	F	df1	df2	Raw P	BH-FDR q
Pnmax	Growth form	21	1.071	1	17	0.3153	0.6021
Pnmax	Leaf habit	21	0.198	1	17	0.6618	0.8332
Pnmax	Growth form × leaf habit	21	0.284	1	17	0.6009	0.822
AQY	Growth form	21	0.152	1	17	0.7014	0.8332
AQY	Leaf habit	21	0.251	1	17	0.6228	0.822
AQY	Growth form × leaf habit	21	1.16	1	17	0.2965	0.6021
LSP	Growth form	19	0	1	15	0.9868	0.9868
LSP	Leaf habit	19	1.433	1	15	0.2499	0.589
LSP	Growth form × leaf habit	19	0.014	1	15	0.9069	0.9655
LCP	Growth form	21	1.092	1	17	0.3107	0.6021
LCP	Leaf habit	21	0.053	1	17	0.8214	0.9036
LCP	Growth form × leaf habit	21	2.634	1	17	0.123	0.4505
SPAD value	Growth form	21	0.37	1	17	0.5512	0.822
SPAD value	Leaf habit	21	4.421	1	17	0.0507	0.2981
SPAD value	Growth form × leaf habit	21	2.885	1	17	0.1076	0.4439
LDMC	Growth form	21	0.896	1	17	0.357	0.6046
LDMC	Leaf habit	21	0.074	1	17	0.7889	0.8977
LDMC	Growth form × leaf habit	21	4.277	1	17	0.0542	0.2981
SLA	Growth form	21	4.781	1	17	0.043	0.2981
SLA	Leaf habit	21	12.729	1	17	0.0024	0.0391
SLA	Growth form × leaf habit	21	14.134	1	17	0.0016	0.0391
LA	Growth form	21	0.146	1	17	0.707	0.8332
LA	Leaf habit	21	2.271	1	17	0.1502	0.4505
LA	Growth form × leaf habit	21	2.406	1	17	0.1393	0.4505
Carea	Growth form	21	0.861	1	17	0.3664	0.6046
Carea	Leaf habit	21	4.419	1	17	0.0508	0.2981
Carea	Growth form × leaf habit	21	0.282	1	17	0.6021	0.822
Narea	Growth form	21	1.012	1	17	0.3284	0.6021
Narea	Leaf habit	21	3.453	1	17	0.0806	0.3797

Narea	Growth form × leaf habit	21	1.673	1	17	0.2132	0.5411
C:N	Growth form	21	0.006	1	17	0.939	0.9684
C:N	Leaf habit	21	0.637	1	17	0.4358	0.6848
C:N	Growth form × leaf habit	21	1.974	1	17	0.1781	0.4897

Notes: The three term-specific P values for each of the 11 response variables were adjusted together using the Benjamini–Hochberg false discovery rate procedure (33 tests). LA was log10-transformed before parametric analyses. For LSP, n = 19 because two species with undefined LSP estimates were excluded.
